# Arthroscopic capsular release is more effective in pain relief than conservative treatment in patients with frozen shoulder

**DOI:** 10.1186/s12891-024-07275-7

**Published:** 2024-02-16

**Authors:** Gábor Skaliczki, Krisztián Kovács, Imre Antal, Imre Sallai, Beáta Kovács, Zoltán Nyőgér, Áron Géresi, Balázs Kiss, Anna Várnagy

**Affiliations:** 1 Department of Orthopedics, Semmelweis University, Korányi Sándor utca 2, Budapest, 1083 Hungary; 2 Department of Biophysics and Radiation Biology, Semmelweis University, Tűzoltó utca 37-47, Budapest, 1094 Hungary; 3 Department of Orthopedics and Traumatology, Petz Aladár University Teaching Hospital, Vasvári Pál utca 2-4, Győr, 9024 Hungary

**Keywords:** Frozen shoulder, Idiopathic stiff shoulder, Treatment, Shoulder, Pain

## Abstract

**Background:**

Frozen shoulder is a common medical condition, but the ideal therapeutic method is yet to be determined. Our aim was to analyze the pain-relieving effect of different treatment options used for the management of this disease.

**Methods:**

Medical records of 59 patients (22 male, 37 female, average age: 55.5 years ±9.9) with early stage primary frozen shoulder were evaluated, their demographic data, physical examination, concomitant diseases and treatment specific data were registered. Life quality and the level of pain were assessed using the Oxford Shoulder Score (OSS) and Numeric Rating Scale (NRS). Different treatment modalities and their effect on pain relief were recorded. Any existing correlation between life quality, pain and demographic data, concomitant diseases or the therapeutic method used was investigated.

**Results:**

The level of pain measured on NRS improved from 7.9 ± 1.6 to 1.9 ± 2.2. The most effective therapeutic method in terms of pain relief was surgery, followed by physiotherapy and intraarticular steroid injection (NRS score after treatment: 2 - *p* < 0.0001; 3.3 - *p* < 0.0001; 4.9 - *p* < 0.0001, respectively). Non-steroidal anti-inflammatory drugs (NSAIDs) did not reduce pain significantly. OSS improved from 24 to 43.6 and was not affected by the investigated variables, time to recovery was not influenced by the demographic data, the type of treatment or concomitant diseases.

**Conclusions:**

Arthroscopic capsular release, physiotherapy and intraarticular steroid injection outperformed physical therapy and NSAID treatment in terms of pain relief. Despite of slight but persistent post-therapeutic pain found in half of the cases, treatment was considered satisfactory by the patients. Nor patient specific neither therapy specific data had a significant effect on the course of the disease.

**Supplementary Information:**

The online version contains supplementary material available at 10.1186/s12891-024-07275-7.

## Introduction

Although the clinical condition described by pain, stiffness and decreased range of motion (ROM) of the glenohumeral joint is well-known and treated frequently, there is still no consensus on the definition, treatment or the name of this disorder. The International Society of Arthroscopy, Knee Surgery and Orthopedic Sports Medicine (ISAKOS) published its definition and classification of the disease in 2014 defining the term “frozen shoulder” as idiopathic stiff shoulder [1] and proposed new criteria for primary and secondary frozen shoulder. This latter nomenclature was used throughout our study.

Based on previous publications, frozen shoulder is considered to have a self-limiting nature that follows a three-phase model [2, 3], however, in the lack of enough supporting evidence recent articles questioned both the path to self-resolution and the phased approach [4, 5].

Treatment of frozen shoulder concentrates on reducing pain and increasing range of motion. Multiple conservative (physiotherapy, non-steroidal anti-inflammatory drugs - NSAIDs, oral steroids, steroid injections, etc.) and surgical (hydrodilatation, mobilization under anesthesia, arthroscopic or open release) therapies are available, although a widely accepted, standardized therapeutic protocol has not been created yet. Usually a set of conservative methods are used, followed by surgery if non-operative treatment fails.

Since pain contributes fundamentally to the deterioration of life quality, our aim was to evaluate the pain-relieving effect of different therapeutic modalities used in the treatment of early stage primary frozen shoulder. Any probable impact of the used therapy on the course of the disease was also registered.

## Materials and methods

The procedures used in this study adhere to the tenets of the Declaration of Helsinki. Patients treated for frozen shoulder at the Department of Orthopedics of the Semmelweis University between November 2011 and October 2019 were enrolled in our retrospective study. Inclusion criteria consisted of the following: diagnosis of primary frozen shoulder according to the ISAKOS 2014 criteria [1], early stage disease (painful shoulder without decrease in the level of pain from the onset of the disease). Exclusion criteria were: previous injury of the shoulder, previous surgery of the shoulder, any specific disorder on plain radiograph, neurological condition that could interfere with the shoulder function, non-compliant patient. After reviewing the charts, 59 patients (59 shoulders) were eligible, all of them gave their consent. Patient specific data (age, gender, concomitant diseases, smoking habit) was registered, the type, frequency and length of treatment was also recorded. The following therapies were used: per oral non-steroid anti-inflammatory drugs (NSAID), intraarticular steroid injection (40 mg triamcinolone acetonide and 4 ml 1% lidocaine), physical therapy (irradiation, ultrasound, electrotherapy, shockwave therapy), physiotherapy (autostretching exercises and soft tissue mobilization). If the patient was unsatisfied with the results after a minimum of 3 months of conservative treatment, arthroscopic capsular release (anterior release without inferior capsular release) was offered. Pain assessment was performed by Numeric Rating Scale (NRS), with a range between 0 and 10, quality of life was measured using the Oxford Shoulder Score (OSS), both questionnaires were filled out by the patients alone. Results of conservative therapy were evaluated 3 months after the initiation, for those patients who required surgery, pain scores and OSS were assessed 6 months after the procedure. The efficiency of each treatment method was evaluated by post-therapy changes in the level of pain and OSS. Any potential connection between the course of the disease, the effect of different treatment modalities and patient specific data was analyzed.

### Statistical analysis

Statistical analyses were performed by Microsoft Excel 2016 and GraphPad Prism 6 or 7 (GraphPad Software, Inc.). Descriptive statistical results are shown as mean ± standard deviation unless stated otherwise. Error bars on the graphs represent the standard error of the mean (SEM). Differences between groups were considered to be statistically significant at a probability value of *p* < 0.01. Mann-Whitney U test was used to compare differences between two independent groups of ordinal dependent variables (OSS comparison between Pre-therapy and Post-therapy groups, NRS comparison between Pre-therapy and Residual pain groups as well as NRS and OSS assessment between Unhealed and Healed groups). One-way ANOVA using Bonferroni post-hoc analysis was performed to assess differences between multiple groups (NRS pain score comparison between Pre-therapy, NSAID, Intraarticular steroid, Physiotherapy, Physicotherapy and Surgery groups). The detailed statistical analysis is summarized in Supplementary Table 3.

## Results

### General results

22 men (37%) and 37 women (63%) participated in our study, the mean age was 55.5 years (± 9.9 years). Comorbidities were registered in 54% (*n* = 32) of our patients, 17 (53%) of those had hypertension, 10 had (31%) thyroid disorder, 7 (22%) were diabetic. Fourteen (24%) patients were smokers.

**Fig. 1:**
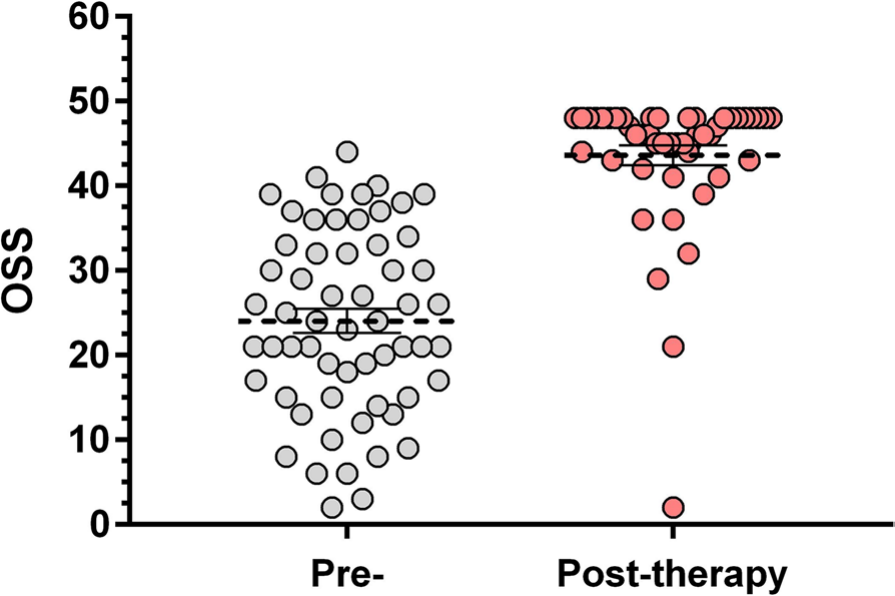
Pre- and post-therapy OSS values (*: *p* < 0.0001, horizontal lines: mean and SEM)

The highest pain at the onset of the disease reached 7.9 ± 1.6 on average (median: 8) measured on NRS, which decreased significantly to 1.9 (±2.2, median:2, *p* < 0.0001) after the therapy. The initial mean value of OSS was 24 (±10.9, median: 24) with a significant improvement to 44 (±8.3, median: 46, *p* < 0.0001) by the end of the treatment (Fig. 1, Table 1) Interestingly, we did not find any correlation between the used treatment modalities or other observed variables and the length of the disease. The mean duration of symptoms subsequent to the onset of the therapy was 19 month (±13.5, median: 17 months) and was independent of the patients’ gender, age, concomitant diseases, smoking habit (*p* = 0.9653, *p* = 0.6514, *p* = 0.7226, *p* = 0.2394, respectively, Mann Whitney test) or of the use of NSAIDS, intraarticular steroid injections or both (*p* = 0.8897, ANOVA test).

**Table 1:** Table showing pre- and post-therapy OSS values

Patient Groups	OSS	
	***Pre-therapy***	***Post-therapy***
Mean	24.03	43.60
Standard Deviation	10.88	8.25
95% CI of the Mean	21.20–26.87	41.21–46.00

### Treatment modalities

Thirty-six percent of our patients (*n* = 21) received oral NSAIDs, the mean duration of the treatment was 3.6 ± 6 months (median: 1 month). NRS mean value did not decrease significantly (from 7.9 ± 1.6 to 7 ± 1.7, *p* = 0.0388) during NSAID therapy and pain recurred in every patient after finishing it.

Intraarticular steroid injection was used in 49% of the cases (*n* = 29), the number of injections given to one patient was 1–8 (mean 2.5, median: 2), the decrease of pain was significant. (NRS value: 4.9 ± 2.2, median: 5, *p* < 0.0001).

Of our patients, 90% (*n* = 53) received physiotherapy for a mean duration of 5.2 months. The exercises were led by a physiotherapist with an average 2.2 times each week (median: 2). After careful education by the physiotherapist, patients performed the exercises at home 5.2 times a week (median: 7). There was a significant reduction of pain after this therapeutical method. (NRS value: 3.3 ± 2.5, median: 3, *p* < 0.0001).

**Fig. 2:**
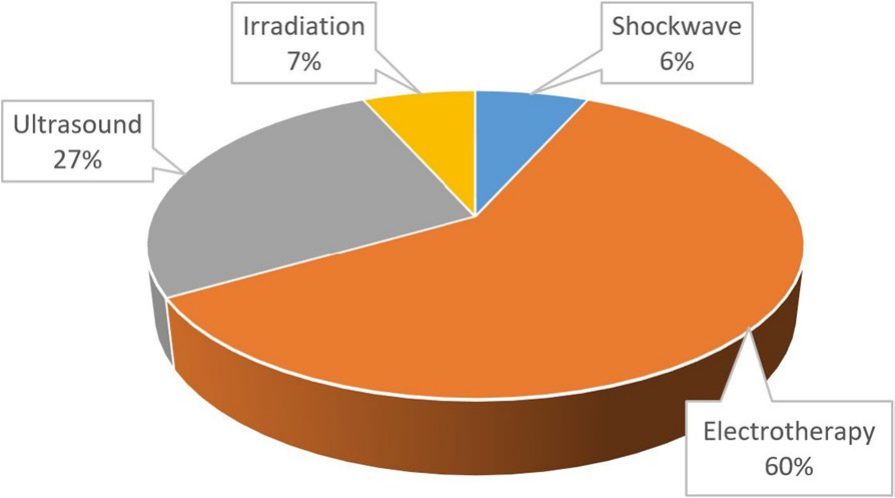
Distribution of different physicotherapeutic methods

Out of 59 patients 15 (25%) participated in physical therapy (electro-, ultrasound, extracorporeal shockwave, and irradiation therapies) which resulted in a mean NRS pain score of 6.8 (median: 7), the improvement of pain was not significant (*p* = 0.029) (Fig. 2).

**Fig. 3:**
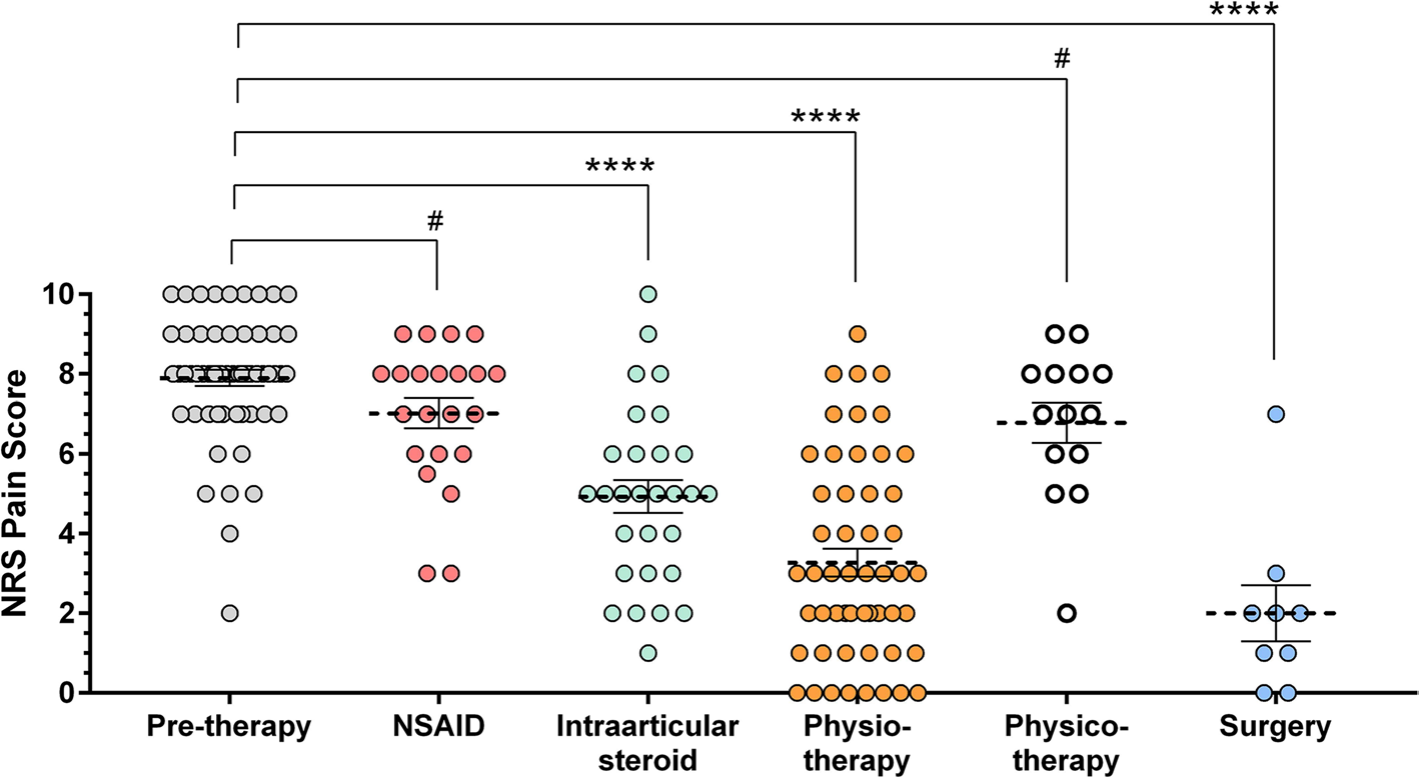
Improvement in the level of pain after different therapeutic methods. Surgery was the most effective followed by physiotherapy and intraarticular steroid injection. NSAID therapy and physical therapy did not provide significant pain relief (#: no significance; ****: significant change, *p* < 0,0001, horizontal lines: mean and SEM)

Surgical management was used in 15.3% of our patients (*n* = 9) after failure of conservative therapy. In all cases arthroscopic capsular release with anteroinferior capsulotomy and release of the coracohumeral ligament and rotator interval was performed, which provided a significant pain relief with a mean NRS pain score of 2 (median 2, *p* < 0.0001) after the procedure (Fig. 3, Table 2).

**Table 2:** NRS pain values in the different therapeutic groups

Patient Groups	NRS Pain Score (rest)					
	***Pre-therapy***	***NSAID***	***Intra-articular steroid***	***Physio- therapy***	***Physico- therapy***	***Surgery***
n	59	22	29	52	14	9
Mean	7.898	7.023	4.931	3.269	6.786	2.000
Standard Deviation	1.561	1.749	2.219	2.529	1.888	2.121
95% CI of the Mean	7.491–8.305	6.247–7.798	4.087–5.775	2.565–3.973	5.695–7.876	0.369–3.631

### Remaining symptoms

**Fig. 4:**
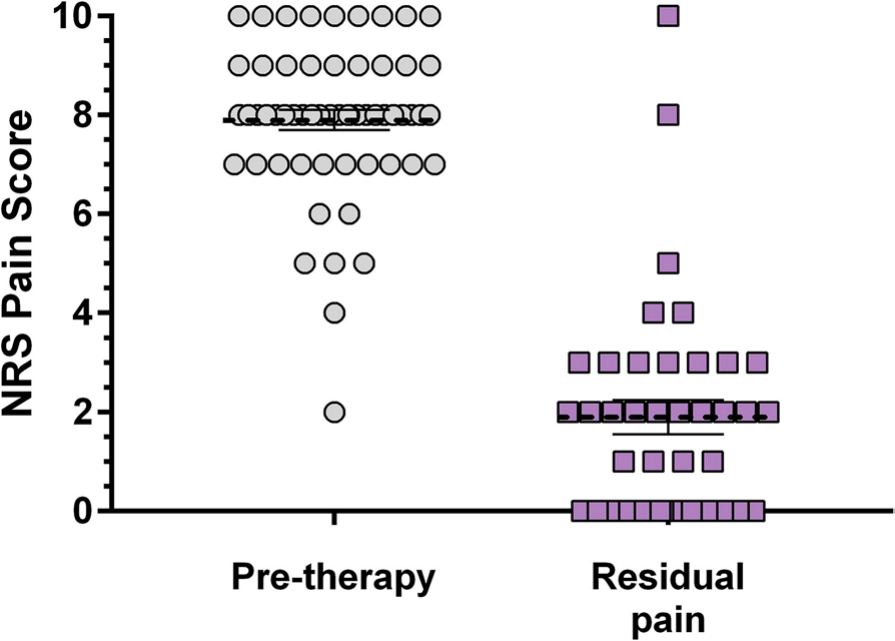
The pre-therapeutic NRS score decreased from 7.9 ± 1.6 (median: 8) to 1.9 ± 2.2 (median:2, *p* < 0.0001, Mann-Whitney test, horizontal lines: mean and SEM)

Our patients were given a question whether they considered themselves as “healed” or “unhealed”. Even though 51% of our patients (*n* = 31) reported a persistent post-therapeutic mild pain, 76.3% (*n* = 45) considered themselves as healed (Fig. 4). Interestingly, the intensity of the post-treatment pain was independent of the therapy used: NSAIDs, corticosteroid injections or the combination of both provided the same pain level at the end of the treatment.

**Fig. 5:**
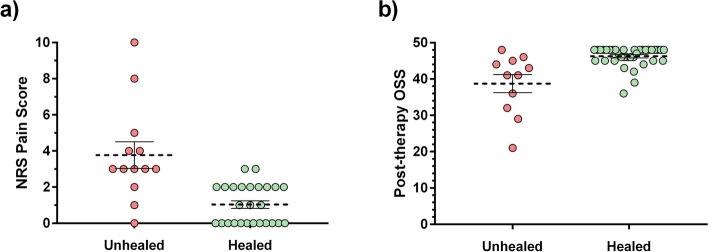
The level of pain among “healed” patients was significantly lower (**a**) and the OSS (**b**) was significantly higher than in the “unhealed” group (*p* < 0.0001 with Mann-Whitney test, horizontal lines: mean and SEM)

There was a significant difference in the measured variables between the group of those who considered themselves healed and those of unhealed. NRS pain score median was 1 in the healed group and 3 in the unhealed group (*p* < 0.0001, Mann Whitney test) while OSS resulted 48 in the healed group and 41 among unhealed patients. (*p* < 0.0001, Mann Whitney test) (Fig. 5).

## Discussion

Despite of numerous studies investigating the disease, a generally used and widely accepted treatment guideline for the treatment and pain management of frozen shoulder is still missing. According to our results arthroscopic capsular release was the most effective in terms of pain relief followed by physiotherapy and intraarticular steroid injection. Physical therapy and NSAID treatment did not decrease pain significantly.

The association between frozen shoulder and endocrine diseases has been shown. Zreik et al. demonstrated that 13.4% of diabetic patients suffer from frozen shoulder, and the incidence of diabetes among patients with frozen shoulder is 30% [6], which is significantly higher than the 8.3% prevalence found in the whole population [7]. This elevated incidence of diabetes was also observed in our study, as 21.9% of our patients were diabetic. Mehta et al. in his study of 42 patients reported significantly worse results after arthroscopic capsulotomy in patients with diabetes then those without, a tendency toward persistent limitation of movement was also observed in the diabetic cohort [8]. This is in contrast to our observation since we did not find any correlation between diabetes and the duration, severity or the course of frozen shoulder. Schiefer et al. found a relationship between the incidence of frozen shoulder and thyroid disorders observing higher prevalence of hypothyroidism among patients with frozen shoulder [9]. The incidence in his cohort of 93 patients was 27.2%, which is similar to the 31.3% prevalence observed in our study. As there is no consensus on the classification of frozen shoulder, it can be debatable if frozen shoulder in a patient with diabetes or thyroid disease is primary or not, however, according to the ISAKOS 2014 classification which was used in our study, frozen shoulder in these cases is considered to be primary [1].

Oral NSAID therapy is widely used in the management of frozen shoulder because NSAIDs are easily available and known to provide excellent pain relief. Unfortunately, they are only useful as symptomatic treatment and their effect was shown to be merely temporary [1, 10, 11]. The same results were also found in our study as pain decreased solely during NSAID therapy and it recurred in every patient after finishing the treatment.

Intraarticular steroid injection is generally accepted as an effective method in the treatment of frozen shoulder. Reported by several studies, it provides better results than NSAIDs in terms of pain relief and increasing ROM [12, 13]. We observed the same as intraarticular steroid injections outperformed NSAIDs in reducing pain. Complications associated with steroids limit their administration in certain patient groups, thus they should only be used with special caution in diabetic patients.

Physiotherapy plays an important role in the conservative management of frozen shoulder, nonetheless its efficiency is controversial. This is probably due to the wide diversity in the type, intensity and frequency of exercises on the one hand, and due to the differences how patients conduct them at home on the other hand. In an effort to minimize these differences, patients in our study were educated thoroughly to perform correctly the exercises at home. Literature data suggests that in the early (painful) phase light exercises combined with intraarticular steroid injections provide satisfactory pain relief, while in later stages intense exercises with auto-stretching are more effective in improving ROM [14]. Our physiotherapy protocol follows these principles, thus it provided a significant pain relief in our study, while – in accordance with other publications – physical therapy did not prove to be efficient [15].

If conservative management fails, surgery is indicated. In our study arthroscopic capsular release was performed after 3 months of unsuccessful conservative treatment. There are several surgical options such as manipulation under anesthesia, hydrodilatation, arthroscopic or open release. The efficiency of arthroscopic release is confirmed by several authors [16, 17], however there is less data on its pain relieving-effect. De Carli et al. observed that in the short-term surgical management provides significantly better function and greater gain in ROM compared to intraarticular steroid injection [18]; long-term benefits of the procedure have also been published [19]. The same results were found in our study, as arthroscopic release proved to be the most efficient method in relieving pain also in the short- and in the long-term.

Residual pain was observed in more than half of our patients (51%), which corresponds with the data published in the international literature [14, 20, 21]. When asking our patients if they considered themselves as “completely healed”, 76,3% answered yes. The most important difference between the “healed” and “unhealed” group was the level of pain, as the NRS pain score was significantly lower in the “healed” group compared to that of the “unhealed” group. It is therefore considerable to focus on pain management in the treatment using methods that provide instant, efficient and long-lasting pain relief.

There are several limitations of our work. Reliable data collection is always a challenge in retrospective studies which limits the number of eligible patients, therefore our cohort is relatively small, which could underpower our statistics. Also, the relatively small number of patients made it impossible to perform subgroup analyzes. Nevertheless, a significant effort was made to teach every patient how to perform the exercises at home, it cannot be guaranteed that all of them conducted physiotherapy perfectly the same way. Different treatment modalities were separated but there could be some overlapping between the groups (i.e. a patient took NSAID a few times after corticosteroid injection) that could bias the results. We can’t rule out with absolute certainty that post-operative pain has affected our results, although 6 months after surgery the chances of this are small.

Based on our results, physical therapy and NSAID treatment did not decrease pain significantly. The most efficient conservative way for pain relief was physiotherapy and intraarticular steroid injection. If conservative therapy failed, arthroscopic capsular release provided excellent pain relief. As pain is probably the most important factor affecting life quality, frozen shoulder therapy should concentrate on pain management.

### Supplementary Information

**Supplementary material 1.**

## Data Availability

The datasets used and/or analysed during the current study available from the corresponding author on request.
