# Supplementary material for: Arthroscopic capsular release is more effective in pain relief than conservative treatment in patients with frozen shoulder
Source: BMC Musculoskelet Disord. 2024 Feb 16;25:145. doi: 10.1186/s12891-024-07275-7 (PMC10870563; doi:10.1186/s12891-024-07275-7)
Supplement: Supplementary file 1 — Supplementary material 1. [file 12891_2024_7275_MOESM1_ESM.docx]

Supplementary Table 3.

Detailed results, statistical methods, statistical values and p values of the different investigated treatment modalities are shown in this supllementary data table.

| **Reference** | **n (data points)** | | **Statistical Method** | **Value of the Test Statistic** | **p-value** |
| --- | --- | --- | --- | --- | --- |
| Fig. 1 | Pre-therapy: 59 | Post-therapy: 48 | Mann-Whitney Test | U=166.5 | p<0.0001 |
| Fig. 3 | Pre-therapy: 59 | | One-way ANOVA | F=48.60 | p<0.0001 |
|  | NSAID: 22 | |  | Comparison with „Pre-therapy” group: mean diff.=0.876 | p=0.4007 |
|  | Intraarticular steroid: 29 | |  | Comparison with „Pre-therapy” group: mean diff.=2.967 | p<0.0001 |
|  | Physiotherapy: 52 | |  | Comparison with „Pre-therapy” group: mean diff.=4.629 | p<0.0001 |
|  | Physicotherapy: 14 | |  | Comparison with „Pre-therapy” group: mean diff.=1.113 | P=0.3289 |
|  | Surgery: 9 | |  | Comparison with „Pre-therapy” group: mean diff.=5.898 | p<0.0001 |
| Fig. 4 | Pre-therapy: 59 | Residual pain: 40 | Mann-Whitney Test | U=102 | p<0.0001 |
| Fig. 5A | Unhealed: 13 | Healed: 26 | Mann-Whitney Test | U=44 | p<0.0001 |
| Fig. 5B | Unhealed: 11 | Healed: 36 | Mann-Whitney Test | U=59 | p<0.0001 |
